# Maternal coffee intake and the risk of bleeding in early pregnancy: a cross-sectional analysis

**DOI:** 10.1186/s12884-020-2798-1

**Published:** 2020-02-21

**Authors:** Hansol Choi, Seul Koo, Hyun-Young Park

**Affiliations:** 1 Department of Epidemiology and Health Index, Center for Genome Science, Korea National Institute of Health, Korea Centers for Disease Control & Prevention, Cheongju, Republic of Korea; 2 Center for Genome Science, Korea National Institute of Health, Korea Centers for Disease Control & Prevention, Cheongju, Republic of Korea

**Keywords:** Caffeine, Coffee consumption, Fetus, Placenta, Pregnancy

## Abstract

**Background:**

Caffeine can easily cross the placenta, and maternal caffeine intake, thus, has an effect on fetal growth. However, it is still unclear whether coffee consumption is an independent risk factor for bleeding in early pregnancy. The objective of this study was to examine the association between pre-pregnancy coffee consumption patterns and the risk of bleeding in early pregnancy.

**Methods:**

A cross-sectional analysis was conducted among 3510 pregnant women from the Korean Pregnancy Outcome Study who underwent baseline examination and for whom the results of the pregnancy were available. Coffee consumption patterns before pregnancy were examined using a questionnaire. The participants were classified according to the frequency of coffee consumption into seldom (< 1 cup/week), light (< 1 cup/day), moderate (1 cup/day), and heavy coffee drinker (≥2 cups/day) groups. Bleeding in early pregnancy was defined as the occurrence of vaginal bleeding in the first 20 weeks of pregnancy. Multiple logistic regression models were applied to examine the association between pre-pregnancy coffee consumption and the risk of bleeding in early pregnancy, after adjusting for age, body mass index (BMI), systolic blood pressure, cigarette smoking and alcohol consumption behavior, previous and current physical activity levels, stress levels, history of depression, antenatal depressive symptoms during the first trimester, type of emesis, parity, and the number of livebirths, stillbirths, miscarriages, and abortions.

**Results:**

Women who were light, moderate, and heavy coffee drinkers before pregnancy had adjusted ORs of 1.086, 1.225, and 1.358, respectively, for bleeding in early pregnancy. In a fully adjusted model, heavy coffee drinkers showed a significantly higher risk of bleeding in early pregnancy, even in women aged 35 years and younger (OR 1.680) and in those with a normal body mass index (OR 1.389), who were at relatively low risk for pregnancy-related complications.

**Conclusions:**

Our results showed that heavy coffee drinking was independently associated with a higher risk of bleeding in early pregnancy among pregnant Korean women, suggesting that caffeine intake before conception and during pregnancy should be reduced. Our study highlights the need for nutritional interventions for healthy coffee drinking among pregnant women in Korea.

## Background

Coffee is one of the most popular beverages worldwide [1–3]. The Korean National Health and Nutrition Examination Survey reported that the prevalence of daily coffee drinking (1 or more cups/day) greatly increased from 54.6% in 2001 to 65.3% in 2010–2011 among Korean adults [4]. The average coffee consumption among Korean adults is 11.3 times/week — more than five times greater than that in other countries in the Asia-Pacific region [3]. Coffee contains several physiologically active substances; caffeine, in particular, is an important component of coffee [5, 6]. Other caffeinated beverages or foods do not contribute significantly to the daily caffeine intake among Koreans [7]. Therefore, it is important to examine the effect of coffee consumption on health.

Interestingly, among all the dietary ingredients with a potential to adversely affect fetoplacental development, caffeine is the most commonly consumed by pregnant women. Maternal caffeine intake during pregnancy affects fetal growth because caffeine can easily cross the placenta and decrease blood flow to the placenta [8, 9]. There are ongoing concerns that coffee intake could increase among pregnant women in particular and result in adverse health effects. However, the specific effects of caffeine on the fetus remain unknown. Moreover, it is still unclear whether coffee consumption is an independent risk factor for bleeding in early pregnancy, which is the most common complication of pregnancy (noted in 15–20% of all ongoing pregnancies) [10, 11] and may indicate underlying placental dysfunction that could induce complications in later phases of pregnancy [12, 13].

Therefore, the aim of this study was to examine pre-pregnancy coffee consumption patterns and their association with the risk of bleeding in early pregnancy among pregnant Korean women.

## Methods

### Study participants

Data for the present study were derived from the Korean Pregnancy Outcome Study (KPOS), a prospective cohort study. Between March 2013 and January 2017, all pregnant women who visited Cheil General Hospital and CHA Hospital for antenatal care during the first trimester were asked to participate in the KPOS. Women were excluded from enrolment if they were not Korean or were pregnant with triplets or higher-order multiple gestations. Gestational age was determined based on the date of the last menstrual period in women who had conceived naturally, and was confirmed by the first trimester ultrasound. After the first antenatal visit, eligible participants were requested to complete several sets of questionnaires or examinations at each of the following visits: visit 1 in the first trimester (around 12 weeks of gestation); visit 2 in the second trimester (around 24 weeks of gestation); visit 3 in the third trimester (around 36 weeks of gestation); visit 4 at birth; and visit 5 at 4–6 weeks after birth.

As shown in Supplementary Fig. 1 [see Additional file 1], after excluding 55 individuals with missing dietary data, we performed a cross-sectional analysis of 3510 women who had positive pregnancy results. Trained research nurses explained the study in detail, obtained written informed consents, and completed questionnaires. All participants provided written informed consent, and the study protocol was approved by the Institutional Review Board (IRB) of Cheil General Hospital (IRB number: CGH-IRB-2013-10) and CHA University Gangnam CHA Hospital IRB (IRB number: 2013–14-KNC13–018), separately. It was clearly explained to all participants that they were free to withdraw from any part of the study at any point in time.

### Measurements

A face-to-face interview was conducted to evaluate participants’ socio-demographic profiles, medical and family history, reproductive information, health-related behaviors, and psychological health. Data on socio-demographic status included age, educational level, household income, employment status, marital status, cohabiting family composition, and information on spouses. Family history of hypertension, diabetes, gestational diabetes mellitus, preeclampsia, depression, and other mental illness was also taken. The questionnaires which we used in this study was uploaded as Supplementary File 1.

Participants underwent clinical and laboratory examinations, including anthropometric measurements, blood pressure measurements, and blood and urine laboratory tests during pregnancy. Asian classifications of obesity were made in this study using the body mass index (BMI) [13]. Symptoms of depression were assessed using the Korean version of the Edinburgh Postnatal Depression Scale (K-EPDS), which is a reliable measurement for peripartum depression and validated questionnaire with 10 items; those with K-EPDS scores ≥10 were considered to have symptoms of antenatal depression [14, 15]. Those taking anti-depressant drugs and those with a self-reported physician’s diagnosis of depression were considered to have a history of depression. Cigarette smoking, alcohol intake, and supplement intake were evaluated during each visit. Physical activity was assessed during each visit with a self-reported questionnaire.

Dietary intake patterns were evaluated using a questionnaire during the first visit. The coffee consumption pattern before conception was determined through the question, “How often did you drink coffee before the pregnancy?” on the questionnaire. Coffee consumption was categorized into five groups (seldom, 2–3 cups/week, 4–6 cups/week, 1 cup/day, and 2 or more cups/day). In the analysis, participants were divided into four groups based on their reported amount of coffee consumption: ≥2 cups/day, “heavy coffee drinkers”; 1 cup/day, “moderate coffee drinkers”; < 1 cup/day, “light coffee drinkers”; and < 1 cup/week, “seldom coffee drinkers” (reference group). Preferences for the following types of coffee were noted: black coffee, black coffee with sugar, black coffee with creamer, and instant coffee mix (instant coffee with creamer and sugar).

We obtained information on antenatal pregnancy complications and birth details. First trimester complications, including emesis and bleeding in early pregnancy, were assessed during the first visit. In this study, bleeding in early pregnancy was defined as the occurrence of vaginal bleeding of a closed cervix in the first 20 weeks of pregnancy, confirmed using ultrasonographic examinations by a physician [16, 17]. The birth outcomes included gestational age at birth, type of labor (induced or spontaneous), type of birth, indication for Caesarean birth, and birth complications.

Blood pressure was measured during every visit using the automatic oscillometric technique, but a diagnosis of hypertensive disorders of pregnancy was confirmed by manual measurements using blood pressure cuffs and auscultation. Blood samples and placenta were stored in − 70 °C freezers at a controlled temperature and humidity. All biological samples were marked with barcodes and stored in the National Biobank of Korea. We uploaded the data from all questionnaires and examinations to a web-based clinical data management system (iCReaT) managed by the Korea National Institute of Health.

### Statistical analysis

We summarized the general characteristics of study participants using means and standard deviations for continuous variables and observed numbers and percentages for categorical variables. To statistically analyze differences among groups, a general linear model and the chi-square test were used for continuous and categorical variables, respectively. The Bonferroni post-hoc test was used to identify groups showing significant differences and the results are shown in Supplementary Fig. 2. For some analyses, the lower categories of exposure variables were combined into a single stratum because of the small number of subjects in these categories. Multivariate logistic regression analysis was used to estimate odds ratios (ORs) with 95% confidence intervals (CIs) for the association between coffee consumption and bleeding in early pregnancy. Age, BMI, systolic blood pressure, cigarette smoking and alcohol consumption behavior, previous and current physical activity levels, stress levels, history of depression, presence of antenatal depressive symptom during the first trimester, type of emesis, parity, and the number of livebirths, stillbirths, miscarriages, and abortions were considered as covariates in the adjusted model.

An additional sensitivity analysis was performed with stratification according to age and BMI. We also performed an additional multiple logistic regression analysis to estimate the association between the type of coffee preferred and the risk of bleeding in early pregnancy. All statistical analyses were performed using the SAS software (version 9.4, SAS; NC, USA), and two-sided *p*-values less than 0.05 were considered indicators of statistical significance.

## Results

**Fig. 1:**
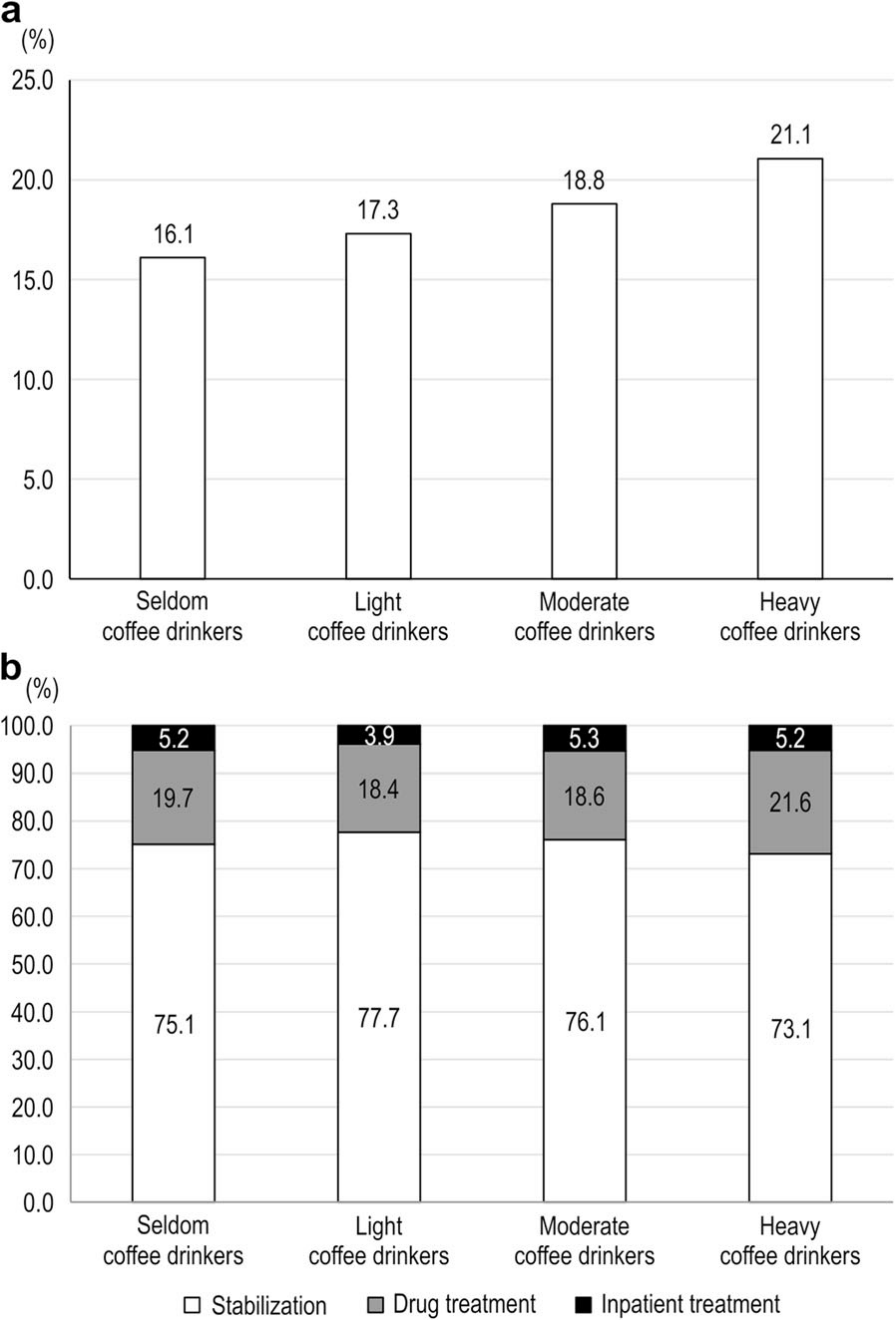
Prevalence and severity of bleeding in early pregnancy according to coffee consumption

Table 1 presents the baseline characteristics of all study participants according to the frequency of coffee consumption before pregnancy. Of the 3510 participants, 1077 were seldom coffee drinkers (30.7%), 595 were light coffee drinkers (17.0%), 1202 were moderate coffee drinkers (34.2%), and 636 were heavy coffee drinkers (18.1%). The mean age of all pregnant women was 33.3 years. Heavy coffee drinkers were more likely to be significantly older; have a higher economic status, BMI, and frequency of history of depression; and be former smokers and drinkers. The overall prevalence of bleeding in early pregnancy among these pregnant women was 18.1%. As shown in Supplementary Table 1, a total of 46 miscarriages or abortions occurred; however, it was not significantly different according to coffee consumption. As shown in Fig. 1, heavy coffee drinkers showed the highest prevalence of bleeding in early pregnancy; this group, in particular, tended to require drug therapy or inpatient treatment for bleeding in early pregnancy.

**Table 1:** Baseline characteristics of study participants (*n* = 3510)

Variables	Frequency of coffee consumption				*p-*value
	Seldom coffee drinkers (*n* = 1077)	Light coffee drinkers (< 1 cup/day) (*n* = 595)	Moderate coffee drinkers (1 cup/day) (*n* = 1202)	Heavy coffee drinkers (≥2 cups/day) (*n* = 636)	
Maternal age, years	32.8 ± 3.8	33.1 ± 3.8	33.4 ± 3.6	34.1 ± 3.8	< 0.001
20–24	11(1.0)	7(1.2)	5(0.4)	1(0.2)	< 0.001
25–29	203(18.8)	99(16.6)	184(15.3)	62(9.7)	
30–34	515(47.8)	277(46.6)	558(46.4)	292(45.9)	
35–39	303(28.1)	185(31.1)	392(32.6)	220(34.6)	
≥ 40	45(4.2)	27(4.5)	63(5.2)	61(9.6)	
Marital status					
Married	1055(98.0)	581(97.6)	1184(98.5)	620(97.5)	0.446
Unmarried	22(2.0)	13(2.2)	18(1.5)	15(2.4)	
Divorced/ Widowed/ Separated	0(0.0)	1(0.2)	0(0.0)	1(0.2)	
Educational status					
≤ High school	92(8.5)	54(9.1)	83(6.9)	64(10.1)	0.075
College or university	810(75.2)	442(74.3)	923(76.8)	447(70.3)	
≥ Graduate school	175(16.2)	99(16.6)	196(16.3)	125(19.7)	
Systolic blood pressure, mmHg	113.1 ± 13.1	114.6 ± 14.1	114.1 ± 13.3	114.6 ± 13.0	0.068
BMI, kg/m^2 *^	21.3 ± 3.1	21.8 ± 3.2	21.7 ± 3.0	22.2 ± 3.1	< 0.001
< 18.5	155(14.4)	52(8.7)	116(9.7)	50(7.9)	< 0.001
18.5–22.9	677(62.9)	379(63.7)	772(64.2)	379(59.6)	
23.0–24.9	134(12.4)	81(13.6)	157(13.1)	104(16.4)	
25.0–29.9	91(8.4)	67(11.3)	137(11.4)	86(13.5)	
≥ 30.0	20(1.9)	16(2.7)	20(1.7)	17(2.7)	
Emesis	801(74.4)	447(75.1)	942(78.4)	497(78.1)	0.085
Bleeding in early pregnancy	173(16.1)	103(17.3)	226(18.8)	134(21.1)	0.059
Stabilization	130(75.1)	80(77.7)	172(76.1)	98(73.1)	0.987
Drug treatment	34(19.7)	19(18.4)	42(18.6)	29(21.6)	
Inpatient treatment	9(5.2)	4(3.9)	12(5.3)	7(5.2)	
Number of fetus					
Singleton	1061(98.5)	584(98.2)	1188(98.8)	623(98.0)	0.461
Twin	16(1.5)	11(1.8)	14(1.2)	13(2.0)	
Parity	0.4 ± 0.6	0.5 ± 0.6	0.5 ± 0.6	0.4 ± 0.6	0.005
0	677(62.9)	341(57.3)	681(56.7)	399(62.7)	0.001
1	362(33.6)	223(37.5)	454(37.8)	192(30.2)	
≥ 2	38(3.5)	31(5.2)	67(5.6)	45(7.1)	
History of depression	5(0.5)	3(0.5)	8(0.7)	5(0.8)	0.037
Antenatal depressive symptoms	194(18.0)	113(19.0)	217(18.1)	141(22.2)	0.136
Type of coffee (n = 2394)					
Black coffee		355(60.4)	781(65.9)	402(64.7)	< 0.001
Black coffee with sugar		50(8.5)	96(8.1)	23(3.7)	
Black coffee with non-dairy creamer		28(4.8)	47(4.0)	15(2.4)	
Instant coffee with sugar and non-dairy creamer		155(26.4)	261(22.0)	181(29.1)	
Cigarette smoking					
Never smoked	970(90.1)	546(91.8)	1081(89.9)	538(84.6)	< 0.001
Former smoker	107(9.9)	47(7.9)	121(10.1)	96(15.1)	
Current smoker	0(0.0)	2(0.3)	0(0.0)	2(0.3)	
Alcohol consumption					
Never drank	245(22.7)	129(21.7)	193(16.1)	98(15.4)	< 0.001
Former drinker	830(77.1)	465(78.2)	1009(83.9)	537(84.4)	
Current drinker	2(0.2)	1(0.2)	0(0.0)	1(0.2)	
Physical activity (*n* = 3459)					
Static activity	265(24.9)	132(22.5)	259(21.9)	136(21.8)	< 0.001
Light activity	551(51.8)	352(60.0)	693(58.5)	387(61.9)	
Moderate activity	246(23.1)	103(17.5)	232(19.6)	100(16.0)	
≤ Vigorous activity	1(0.1)	0(0.0)	0(0.0)	2(0.3)	

Data expressed as mean ± standard deviation or number (percentage)

^*^Asian classification of obesity used in this study

**Fig. 2:**
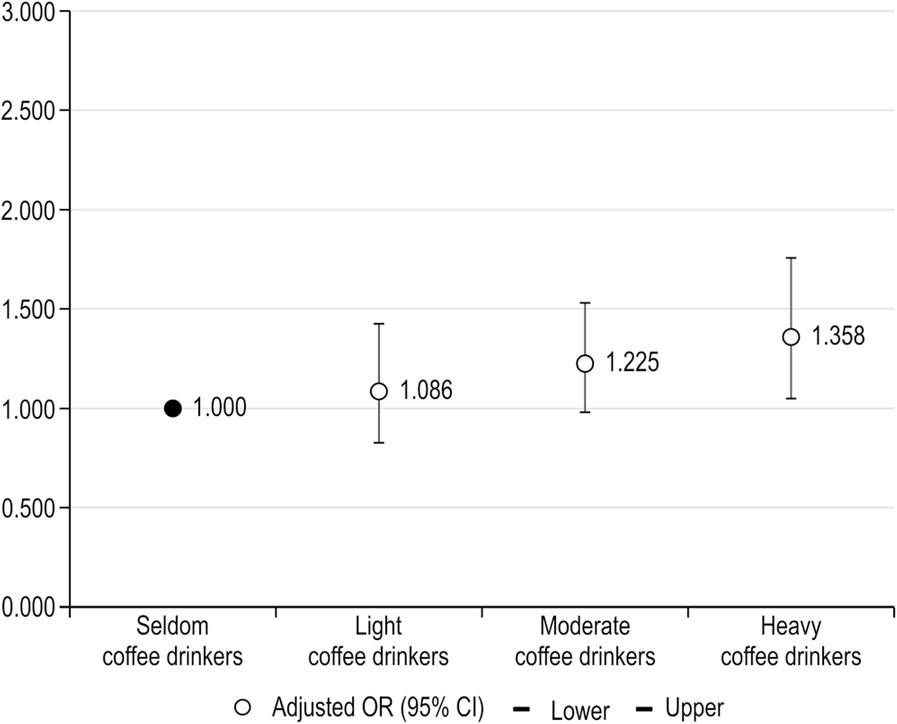
Adjusted odds ratios for the risk of bleeding in early pregnancy according to coffee consumption

Age- and BMI-dependent adjusted ORs for bleeding in early pregnancy in the different coffee consumption groups are presented in Tables 2 and 3, respectively. The group with the highest coffee consumption showed a high risk of bleeding in early pregnancy, and this association was significant both before and after additional adjustment for covariates. As shown in Fig. 2, heavy coffee drinkers showed a significantly higher prevalence of bleeding in early pregnancy than did seldom coffee drinkers. Light, moderate, and heavy coffee drinkers showed adjusted ORs of 1.086, 1.225, and 1.358, respectively, for bleeding in early pregnancy. This association was significant in all age groups, except for in women older than 35–40 years, who are already at risk of pregnancy complications (Table 2 and Supplementary Table 2) (see Additional file 1).

**Table 2:** Association between coffee consumption frequency and the risk of bleeding in early pregnancy according to age (n = 3510)

	Total No.	No. (%)	Unadjusted OR (95% CI)	Adjusted OR (95% CI)^a^
Overall	3510	636(18.1)		
Seldom coffee drinkers	1077	173(16.1)	1.000	1.000
Light coffee drinkers (< 1 cup/day)	595	103(17.3)	1.094(0.837–1.429)	1.086(0.827–1.425)
Moderate coffee drinkers (1 cup/day)	1202	226(18.8)	1.210(0.973–1.504)	1.225(0.981–1.530)
Heavy coffee drinkers (≥2 cups/day)	636	134(21.1)	1.395(1.086–1.792)	1.358(1.050–1.757)
Aged < 35 years	2214	372(16.8)		
Seldom coffee drinkers	729	111(15.2)	1.000	1.000
Light coffee drinkers (< 1 cup/day)	383	57(14.9)	0.973(0.688–1.376)	0.993(0.699–1.411)
Moderate coffee drinkers (1 cup/day)	747	125(16.7)	1.119(0.847–1.479)	1.163(0.875–1.547)
Heavy coffee drinkers (≥2 cups/day)	355	79(22.3)	1.594(1.155–2.198)	1.680(1.207–2.338)
Aged ≥35 years	1296	264(20.4)		
Seldom coffee drinkers	348	62(17.8)	1.000	1.000
Light coffee drinkers (< 1 cup/day)	212	46(21.7)	0.973(0.688–1.376)	1.278(0.834–1.958)
Moderate coffee drinkers (1 cup/day)	455	101(22.2)	1.119(0.847–1.479)	1.316(0.925–1.872)
Heavy coffee drinkers (≥2 cups/day)	281	55(19.6)	1.594(1.155–2.198)	1.123(0.750–1.679)

^a^adjusted for age, body mass index, systolic blood pressure, cigarette smoking and alcohol consumption behavior, previous and current physical activity levels, stress levels, history of depression, presence of antenatal depressive symptoms during the first trimester, type of emesis, parity, and the number of livebirths, stillbirths, miscarriages, and abortions

**Table 3:** Association between coffee consumption frequency and the risk of bleeding in early pregnancy according to BMI (n = 3510)

BMI	Total No.	No. (%)	Unadjusted OR (95% CI)	Adjusted OR (95% CI)^a^
Underweight, < 18.5 kg/m^2^	373	80(21.4)		
Seldom coffee drinkers	155	33(21.3)	1.000	1.000
Light coffee drinkers (< 1 cup/day)	52	11(21.2)	0.992(0.460–2.139)	0.780(0.331–1.837)
Moderate coffee drinkers (1 cup/day)	116	26(22.4)	1.068(0.597–1.911)	1.213(0.650–2.265)
Heavy coffee drinkers (≥2 cups/day)	50	10(20.0)	0.924(0.418–2.042)	0.990(0.414–2.367)
Normal, 18.5–24.9 kg/m^2^	2683	463(17.3)		
Seldom coffee drinkers	811	119(14.7)	1.000	1.000
Light coffee drinkers (< 1 cup/day)	460	80(17.4)	1.224(0.898–1.669)	1.249(0.912–1.712)
Moderate coffee drinkers (1 cup/day)	929	168(18.1)	1.284(0.993–1.659)	1.314(1.012–1.706)
Heavy coffee drinkers (≥2 cups/day)	483	96(19.9)	1.442(1.073–1.940)	1.389(1.025–1.884)
Overweight + Obese, ≥25.0 kg/m^2^	454	93(20.5)		
Seldom coffee drinkers	111	21(18.9)	1.000	1.000
Light coffee drinkers (< 1 cup/day)	83	12(14.5)	0.724(0.334–1.571)	0.699(0.315–1.551)
Moderate coffee drinkers (1 cup/day)	157	32(20.4)	1.097(0.594–2.026)	1.059(0.554–2.025)
Heavy coffee drinkers (≥2 cups/day)	103	28(27.2)	1.600(0.841–3.045)	1.574(0.801–3.093)

^a^adjusted for age, body mass index, systolic blood pressure, cigarette smoking and alcohol consumption behavior, previous and current physical activity levels, stress levels, history of depression, presence of antenatal depressive symptoms during the first trimester, type of emesis, parity, and the number of livebirths, stillbirths, miscarriages, and abortions

Regarding BMI, a higher consumption of coffee was significantly associated with the risk of bleeding in early pregnancy in women with normal BMI (Table 3). However, this association was not statistically significant in overweight or obese pregnant women.

As shown in Supplementary Table 3 [see Additional file 1], no significant associations were observed between the type of coffee, additives, and the risk of bleeding in early pregnancy.

## Discussion

As younger women continue to partake in the western coffee culture and demand high-quality coffee beans, the current trends of coffee consumption are expected to continue. The properties of coffee make it a double-edged sword, and the balance between its beneficial and harmful health impacts should be considered. In this study, we examined coffee consumption patterns before pregnancy and their association with the risk of bleeding in early pregnancy among pregnant Korean women. Our study showed that women who were habitual coffee drinkers before pregnancy constituted a larger fraction of those experiencing bleeding in early pregnancy than women who were seldom coffee drinkers. We found that habitual coffee consumption of one or more cup/day before pregnancy was significantly associated with an increased risk of bleeding in early pregnancy, even after adjustment for cigarette smoking and alcohol consumption. However, the type of coffee consumed did not significantly affect the risk of bleeding in early pregnancy.

In the present study, among 3510 pregnant women, the overall prevalence of bleeding in early pregnancy was 18.1%, even though the average maternal age of participants was relatively high, which is consistent with previous results. Bleeding in early pregnancy is associated with an increased risk of poor fetal and maternal outcomes, and perinatal mortality was observed to be more than twice as frequent in women who experienced bleeding in early pregnancy in the meta-analysis than in those who did not [12]. First trimester bleeding could indicate an underlying placental dysfunction, which may be related to later pregnancy complications [12]. Therefore, thorough prevention and management with healthy behavior from preconception to early pregnancy may help to prevent future fetal mortalities and morbidities. However, information on risk factors for bleeding in early pregnancy in the first trimester is insufficient and it is still unclear whether specific effects of caffeine is an independent risk factor for bleeding in early pregnancy [12, 18–21].

Coffee consumption is one possible risk factor for bleeding in early pregnancy in the first trimester. Even though the market for caffeinated beverages has increased in the past decades, coffee remains the most frequently consumed caffeinated beverage [1, 7]. A standard cup of coffee is generally expected to provide 100 mg of caffeine; however, this varies according to portion size, brewing method, and brand [1–3, 6, 7, 22]. Although other beverages have some caffeine content (one cup of tea, 64.0 mg of caffeine; 12 oz. of coke, 46.0 mg of caffeine; one cup of hot chocolate, 16.0 mg of caffeine; and caffeinated soda, 46.0 mg of caffeine), these caffeinated beverages do not significantly affect daily caffeine consumption among Koreans [7, 23]. Nisenblat et al. reported that caffeine intake is not associated with an increased risk of bleeding in early pregnancy, with the possible exception of very high levels of caffeine intake [22]. However, caffeine and its metabolites easily cross the placenta and may be present in considerable quantities in the amniotic fluid and fetal blood [8, 24]. Moreover, the fetus metabolizes caffeine very slowly, and even extremely small amounts of maternal caffeine intake could lead to long-term fetal caffeine exposure [22, 24]. Experimental and human studies have shown that caffeine exposure induces angiotensin II by stimulating the generation of reactive oxygen species, which ultimately inhibit angiogenesis and negatively affect the developing embryo [25]. In addition, caffeine consumption could increase the generation of circulating catecholamines, which could cause uteroplacental vasoconstriction, leading to fetal hypoxia [22, 26, 27]. Moreover, although a threshold for the adverse effects of caffeine on pregnant women was not well established, a few studies showed that high levels of caffeine intake could have adverse effects, such as miscarriage, fetal growth restriction, and long-term behavioral effects in offspring [9, 11, 16, 22, 23, 28, 29].

Recently, some epidemiologic studies have found a significant association between a caffeine intake of 300 mg or more/day and the risk of early pregnancy loss [23, 30, 31]. Consistent with previous reports, in the present study, we found that pregnant women who were heavy coffee drinkers had a significantly higher risk of bleeding in early pregnancy. In a Chinese prospective study, caffeine intake before pregnancy was not found to increase the risk of early pregnancy loss, but caffeine intake of more than 300 mg/day during the first trimester appeared to significantly increase this risk [23]. A UK case-control study showed that caffeine consumption of more than 300 mg/day during pregnancy approximately doubles the risk of miscarriage, and this effect is driven by coffee consumption [30]. Similarly, a study found that the adjusted risk of early pregnancy loss among Danish women who consumed more than 375 mg of caffeine/day was 2.21 [31].

A meta-analysis found that the risk of pregnancy loss increased by 3% for every increase in coffee consumption of two cups/day [28]. Hence, most women try to reduce their caffeine intake considerably during pregnancy, especially from the time they start preparing for pregnancy to the first trimester [9, 29]. The current guidelines of the World Health Organization recommend a caffeine intake below 300 mg/day, whereas the American College of Obstetricians and Gynecologists recommend a maximum caffeine intake of 200 mg/day [32, 33]. Different recommendations in guidelines can lead to confusion in preparing for pregnancy or during pregnancy. Moreover, knowing the exact caffeine content is difficult, because the amount varies depending on the serving size of the coffee.

The present study has some limitations that should be noted. The main limitation of the present study relates to the inaccurate assessment of the coffee consumption pattern, because the caffeine consumed in a “cup” of coffee varies according to portion size, brewing method, and brand type. Despite the huge popularity of decaffeinated coffee, we could not examine the consumption of decaffeinated coffee, especially in pregnant women. In future studies, objective measurements, combining caffeine exposure biomarkers from blood, urine, and saliva with 24-h dietary recall measurements, should be used to assess precise coffee consumption. Second, recall bias due to the retrospective assessment of caffeine consumption should be considered. However, since we examined coffee consumption before the onset of bleeding in early pregnancy, the impact of recall bias may have reduced. Third, coffee consumption patterns before pregnancy at a single time point may not reflect chronic exposure over the years, because women who prepare to conceive tend to maintain healthy eating habits. To address this concern in future studies, we should measure coffee consumption before pregnancy and evaluate the reliability of the FFQ. Finally, although we controlled for several potential confounders in our analysis, residual confounding by the effects of diet or other lifestyle factors may have been present. Additionally, a causal relationship between coffee consumption and bleeding in early pregnancy could not be assessed due to the observational design of this study.

## Conclusions

Our results revealed a higher risk of bleeding in early pregnancy among those with heavy coffee consumption before pregnancy. Considering that coffee consumption is a potentially modifiable risk factor, our results indicate that caffeine intake before conception and during pregnancy should be reduced. Moreover, our study provides potentially useful information that can be used to address the need for nutritional interventions for healthy coffee drinking among pregnant women in Korea. Accordingly, it is necessary to recommend pregnant women to limit the amount of caffeine intake per day. However, further prospective studies are needed to confirm our findings and establish the causal associations between the potential negative effects of coffee consumption and the risk of bleeding in early pregnancy.

## Supplementary information

**Supplementary Table 1. **Postpartum characteristics of study participants (*n* = 3510)

**Supplementary Table 2. **Association between the frequency of coffee consumption and risk of bleeding in early pregnancy in pregnant women aged < 40 years (*n* = 3314)

**Supplementary Table 3. **Association between the type of coffee consumption and risk of bleeding in early pregnancy (*n* = 2394)

**Supplementary Fig. 1.** Flowchart of participant selection

**Supplementary Fig. 2.** Post-hoc analysis using the Bonferroni test

**Supplementary File 1.** Questionnaires for the KPOS study

## Data Availability

The KPOS is being conducted mainly at the Cheil General Hospital & Women’s Healthcare Center and CHA Gangnam Medical Center, where the staff are responsible for the collection, management, and distribution of data. All data are stored electronically in an anonymous format and are currently only available to KPOS researchers; however, data analysis collaborations may be possible through specific research proposals. Further information can be requested by e-mailing the principal investigator (hmryu2012@naver.com).

## Abbreviations

CI: Confidence intervals
IPAQ: International Physical Activity Questionnaire
IRB: Institutional Review Board
KPOS: Korean Pregnancy Outcome Study
OR: Odds ratios

## References

[1] Mitchell DC, Knight CA, Hockenberry J, Teplansky R, Hartman TJ (2014). Beverage caffeine intakes in the US. Food Chem Toxicol.

[2] Verster JC, Koenig J (2018). Caffeine intake and its sources: a review of national representative studies. Crit Rev Food Sci Nutr.

[3] United States Department of Agriculture and Foreign Agricultural Service. South Korea: Coffee Market Brief Update. 2016. Available from: https://www.fas.usda.gov/data/south-korea-coffee-market-brief-update

[4] Je Y, Jeong S, Park T (2014). Coffee consumption patterns in Korean adults: the Korean National Health and nutrition examination survey (2001-2011). Asia Pac J Clin Nutr.

[5] Ludwig IA, Clifford MN, Lean ME, Ashihara H, Crozier A (2014). Coffee: biochemistry and potential impact on health. Food Funct.

[6] Heckman MA, Weil J (2010). Gonzalez de Mejia E: caffeine (1, 3, 7-trimethylxanthine) in foods: a comprehensive review on consumption, functionality, safety, and regulatory matters. J Food Sci.

[7] Lim HS, Hwang JY, Choi JC, Kim M (2015). Assessment of caffeine intake in the Korean population. Food Addit Contam Part A Chem Anal Control Expo Risk Assess.

[8] Sajadi-Ernazarova KR, Hamilton RJ. Caffeine, Withdrawal. [Updated 2019 Jul 30]. In: StatPearls [internet]. Treasure Island (FL): StatPearls Publishing. 2019. Available from: https://www.ncbi.nlm.nih.gov/books/NBK430790/. 28613541

[9] CARE Study Group (2008). Maternal caffeine intake during pregnancy and risk of fetal growth restriction: a large prospective observational study. BMJ.

[10] Jouppila P (1985). Clinical consequences after ultrasonic diagnosis of intrauterine hematoma in threatened abortion. J Clin Ultrasound.

[11] Weng X, Odouli R, Li DK. Maternal caffeine consumption during pregnancy and the risk of miscarriage: a prospective cohort study. Am J Obstet Gynecol. 2008;198:279.e1–8. 10.1016/j.ajog.2007.10.803 18221932

[12] Saraswat L, Bhattacharya S, Maheshwari A, Bhattacharya S (2010). Maternal and perinatal outcome in women with threatened miscarriage in the first trimester: a systematic review. BJOG.

[13] World Health Organization (WHO). International Association for the Study of Obesity (IASO) and International Obesity Task Force (IOTF). The Asia-Pacific Perspective: Redefining Obesity and its Treatment. World Health Organization. Geneva: Academic; 2000. p. 378–420.

[14] Cox JL, Holden JM, Sagovsky R (1987). Detection of postnatal depression: development of the 10-item Edinburgh postnatal depression scale. Br J Psychiatry.

[15] Kim YK, Hur JW, Kim KH, Oh KS, Shin YC (2008). Clinical application of Korean version of Edinburgh postnatal depression scale. Psychiatry Clin Neurosci.

[16] Johns J, Muttukrishna S, Lygnos M, Groome N, Jauniaux E (2007). Maternal serum hormone concentrations for prediction of adverse outcome in threatened miscarriage. Reprod BioMed Online.

[17] Woodward PJ, Kennedy A, Sohaey R. Diagnostic imaging: obstetrics. 3^rd^ ed. Elsevier Health Sciences; 2016.

[18] Kanmaz AG, Inan AH, Beyan E, Budak A (2019). The effects of threatened abortions on pregnancy outcomes. Ginekol Pol.

[19] Farrell T, Owen P (1996). The significance of extrachorionic membrane separation in threatened miscarriage. Br J Obstet Gynaecol.

[20] Dadkhah F, Kashanian M, Eliasi G (2010). A comparison between the pregnancy outcome in women both with or without threatened abortion. Early Hum Dev.

[21] Van Oppenraaij R, Jauniaux E, Christiansen O, Horcajadas J, Farquharson R, Exalto N (2009). Predicting adverse obstetric outcome after early pregnancy events and complications: a review. Hum Reprod Update.

[22] Nisenblat V, Norman RJ. The effects of caffeine on reproductive outcomes in women. Retrieved October. 2016;16.

[23] Wen W, Shu XO, Jacobs DR, Brown JE (2001). The associations of maternal caffeine consumption and nausea with spontaneous abortion. Epidemiology.

[24] Berger A (1988). Effects of caffeine consumption on pregnancy outcome. A Rev J Reprod Med.

[25] Ma Z, Wang G, Wh L, Cheng X, Chuai M, KKH L, Yang X (2016). Investigating the effect of excess caffeine exposure on placental angiogenesis using chicken ‘functional’ placental blood vessel network. J Appl Toxicol.

[26] Kirkinen P, Jouppila P, Koivula A, Vuori J, Puukka M (1983). The effect of caffeine on placental and fetal blood flow in human pregnancy. Am J Obstet Gynecol.

[27] Tsubouchi H, Shimoya K, Hayashi S, Toda M, Morimoto K, Murata Y (2006). Effect of coffee intake on blood flow and maternal stress during the third trimester of pregnancy. Int J Gynecol Obstet.

[28] Li J, Zhao H, Song JM, Zhang J, Tang YL, Xin CM (2015). A meta-analysis of risk of pregnancy loss and caffeine and coffee consumption during pregnancy. Int J Gynaecol Obstet.

[29] Peck JD, Leviton A, Cowan LD (2010). A review of the epidemiologic evidence concerning the reproductive health effects of caffeine consumption: a 2000–2009 update. Food Chem Toxicol.

[30] Giannelli M, Doyle P, Roman E, Pelerin M, Hermon C (2003). The effect of caffeine consumption and nausea on the risk of miscarriage. Paediatr Perinat Epidemiol.

[31] Rasch V (2003). Cigarette, alcohol, and caffeine consumption: risk factors for spontaneous abortion. Acta Obstet Gynecol Scand.

[32] American College of Obstetricians and Gynecologists (2010). Moderate caffeine consumption during pregnancy. Committee Opinion No. 462. Obstet Gynecol.

[33] World Health Organization. The world health report 2002: reducing risks, promoting healthy life. WHO press kit; 2002.

