# Supplementary material for: Maternal coffee intake and the risk of bleeding in early pregnancy: a cross-sectional analysis
Source: BMC Pregnancy Childbirth. 2020 Feb 21;20:121. doi: 10.1186/s12884-020-2798-1 (PMC7035749; doi:10.1186/s12884-020-2798-1)
Supplement: Supplementary file 1 — Supplementary Table 1. Postpartum characteristics of study participants (n = 3510) [file 12884_2020_2798_MOESM1_ESM.docx]

| **Supplementary Table 1. Postpartum characteristics of study participants (n=3510)** | | | | | | | | | | | | | | |
| --- | --- | --- | --- | --- | --- | --- | --- | --- | --- | --- | --- | --- | --- | --- |
| Variables | Frequency of coffee consumption | | | | | | | | | | | | | *p-*value |
|  | Seldom coffee drinkers (n=1077) | | Light coffee drinkers (<1 cup/day) (n=595) | | Moderate coffee drinkers (1 cup/day) (n=1202) | | | | | Heavy coffee drinkers (≥2 cups/day) (n=636) | | | |  |
| Result of pregnancy |  |  |  |  |  | | |  | |  | |  | |  |
| Miscarriage/abortion | 10 | (0.9) | 4 | (0.7) | 7 | | | (0.6) | | 9 | | (1.4) | | 0.724 |
| Stillbirth | 5 | (0.5) | 3 | (0.5) | 7 | | | (0.6) | | 1 | | (0.2) | |  |
| Birth | 1062 | (98.6) | 588 | (98.8) | 1188 | | | (98.8) | | 626 | | (98.4) | |  |
| Gestational age at birth, weeks (n=3464) | |  |  |  |  | | |  | |  | |  | |  |
| Pre-term (<37) | 59 | (5.5) | 29 | (4.9) | 65 | | | (5.4) | | 38 | | (6.0) | | 0.892 |
| Normal-term (37-41) | 1002 | (93.0) | 559 | (93.9) | 1122 | | | (93.3) | | 588 | | (92.5) | |  |
| Post-term (≥42) | 1 | (0.1) | 0 | (0.0) | 1 | | | (0.1) | | 0 | | (0.0) | |  |
| Type of birth |  |  |  |  |  | | |  | |  | |  | |  |
| Vaginal | 648 | (60.2) | 350 | (58.8) | 713 | | | (59.3) | | 344 | | (54.1) | | 0.091 |
| Cesarean | 414 | (38.4) | 238 | (40.0) | 475 | | | (238.0) | | 282 | | (44.3) | |  |
| Postpartum depressive symptoms | 96 | (8.9) | 89 | (15.0) | 139 | | | (11.6) | | 94 | | (14.8) | | <0.001 |
| Complications at birth | 122 | (11.3) | 60 | (10.1) | 149 | | | (12.4) | | 72 | | (11.3) | | 0.541 |
| Injuries of parturient canal | 45 | (4.2) | 17 | (2.9) | 42 | | | (3.5) | | 21 | | (3.3) | | 0.646 |
| Placental rupture | 4 | (0.4) | 2 | (0.3) | 1 | | | (0.1) | | 2 | | (0.3) | |  |
| Premature rupture of membranes | 65 | (6.0) | 34 | (5.7) | 97 | | | (8.1) | | 43 | | (6.8) | |  |
| Rupture of uterus/Eclampsia/etc. | 8 | (0.7) | 7 | (1.2) | 9 | | | (0.7) | | 6 | | (0.9) | |  |
| Data expressed as mean ± standard deviation or number (percentage) | | | | | |  |  | |  | |  | |  |  |
