# Supplementary material for: Maternal coffee intake and the risk of bleeding in early pregnancy: a cross-sectional analysis
Source: BMC Pregnancy Childbirth. 2020 Feb 21;20:121. doi: 10.1186/s12884-020-2798-1 (PMC7035749; doi:10.1186/s12884-020-2798-1)
Supplement: Supplementary file 2 — Supplementary Table 2. Association between the frequency of coffee consumption and risk of bleeding in early pregnancy in pregnant women aged < 40 years (n = 3314) [file 12884_2020_2798_MOESM2_ESM.docx]

| **Supplementary Table 2. Association between the frequency of coffee consumption and risk of bleeding in early pregnancy in pregnant women aged <40 years (n=3314)** | | | | | | | | | | | |
| --- | --- | --- | --- | --- | --- | --- | --- | --- | --- | --- | --- |
|  | Total No. | No. (%) | | Unadjusted OR  (95% CI) | | | | Adjusted OR  (95% CI)^a^ | | | |
| Aged <40 years | 3314 | 590 | (17.8) |  |  |  |  |  |  |  |  |
| Seldom coffee drinkers | 1032 | 163 | (15.8) | 1.000 |  |  |  | 1.000 |  |  |  |
| Light coffee drinkers (<1 cup/day) | 568 | 96 | (16.9) | 1.084 | (0.823 | - | 1.429) | 1.090 | (0.823 | - | 1.443) |
| Moderate coffee drinkers (1 cup/day) | 1139 | 215 | (18.9) | 1.240 | (0.992 | - | 1.551) | 1.267 | (1.008 | - | 1.592) |
| Heavy coffee drinkers (≥2 cups/day) | 575 | 116 | (20.2) | 1.347 | (1.035 | - | 1.754) | 1.353 | (1.033 | - | 1.772) |
| ^a^adjusted for age, body mass index, systolic blood pressure, cigarette smoking and alcohol consumption behavior, previous and current physical activity levels, stress levels, history of depression, presence of antenatal depressive symptoms during the first trimester, type of emesis, parity, and the number of livebirths, stillbirths, miscarriages, and abortions | | | | | | | | | | | |
