# Supplementary material for: Maternal coffee intake and the risk of bleeding in early pregnancy: a cross-sectional analysis
Source: BMC Pregnancy Childbirth. 2020 Feb 21;20:121. doi: 10.1186/s12884-020-2798-1 (PMC7035749; doi:10.1186/s12884-020-2798-1)
Supplement: Supplementary file 3 — Supplementary Table 3. Association between the type of coffee consumption and risk of bleeding in early pregnancy (n = 2394) [file 12884_2020_2798_MOESM3_ESM.docx]

| **Supplementary Table 3. Association between the type of coffee consumption and risk of bleeding in early pregnancy (n=2394)** | | | | | | | | | | | | |
| --- | --- | --- | --- | --- | --- | --- | --- | --- | --- | --- | --- | --- |
|  | Total No. | No. (%) | | Unadjusted OR  (95% CI) | | | | Adjusted OR  (95% CI)^a^ | | | | |
| Black coffee | 1538 | 292 | (19.0) | 1.000 |  |  |  | | 1.000 |  |  |  |
| Black coffee with sugar | 169 | 43 | (25.4) | 1.457 | (1.007 | - | 2.079) | | 1.446 | (0.992 | - | 2.107) |
| Black coffee with non-dairy creamer | 90 | 13 | (14.4) | 0.721 | (0.395 | - | 1.737) | | 0.694 | (0.378 | - | 1.277) |
| Instant coffee | 597 | 108 | (18.1) | 0.942 | (0.738 | - | 2.533) | | 0.893 | (0.694 | - | 1.151) |
| ^a^adjusted for age, body mass index, systolic blood pressure, cigarette smoking and alcohol consumption behavior, previous and current physical activity levels, stress levels, history of depression, presence of antenatal depressive symptoms during the first trimester, type of emesis, parity, and the number of livebirths, stillbirths, miscarriages, and abortions | | | | | | | | | | | | |
