# Supplementary figures and images for: Maternal coffee intake and the risk of bleeding in early pregnancy: a cross-sectional analysis
Source: BMC Pregnancy Childbirth. 2020 Feb 21;20:121. doi: 10.1186/s12884-020-2798-1 (PMC7035749; doi:10.1186/s12884-020-2798-1)

**
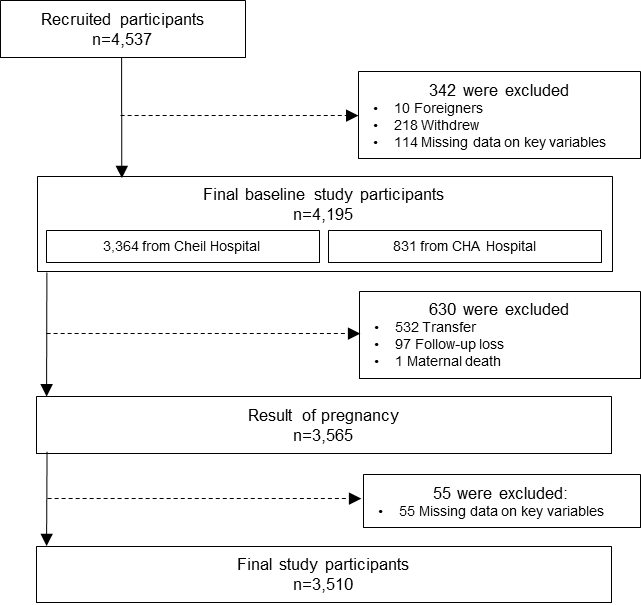
**

**Supplementary Figure 1. Flowchart of participant selection**

Supplement: Supplementary file 4 — Supplementary Fig. 1. Flowchart of participant selection [file 12884_2020_2798_MOESM4_ESM.docx]
