# Supplementary material for: Maternal coffee intake and the risk of bleeding in early pregnancy: a cross-sectional analysis
Source: BMC Pregnancy Childbirth. 2020 Feb 21;20:121. doi: 10.1186/s12884-020-2798-1 (PMC7035749; doi:10.1186/s12884-020-2798-1)

**Supplementary Figure 2. Post-hoc analysis using the Bonferroni test
Variable: Age**


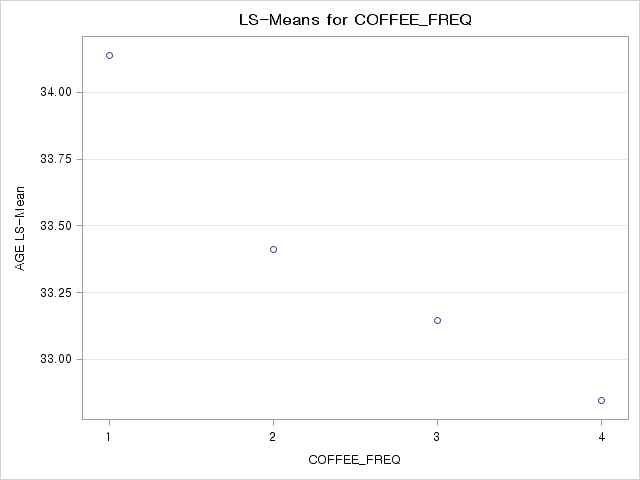


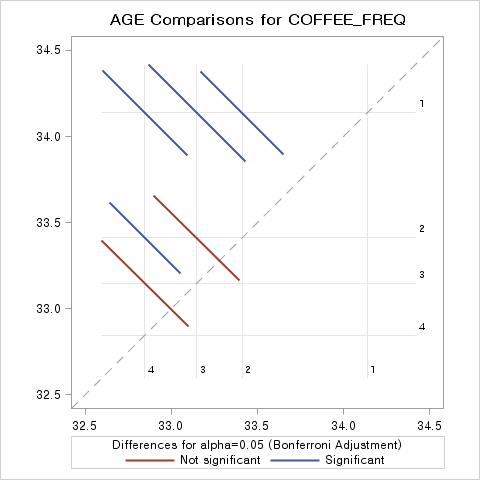


**Variable: Systolic blood pressure**


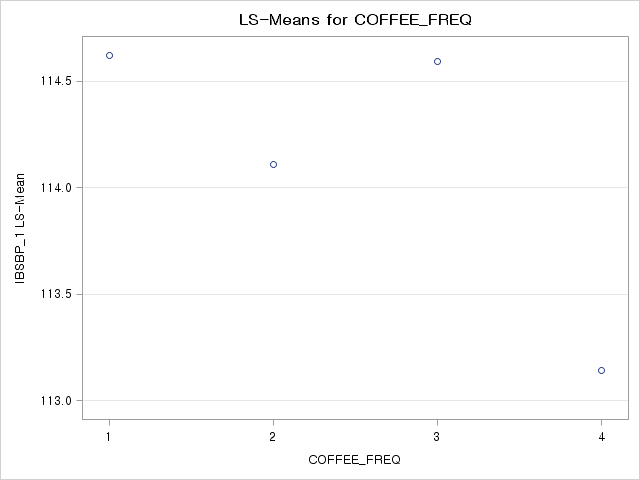

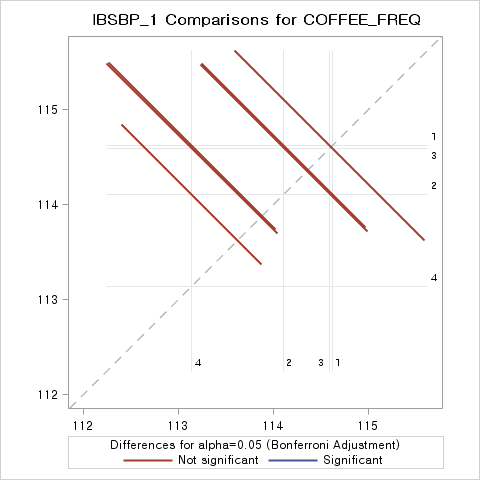


**Variable: Diastolic blood pressure**


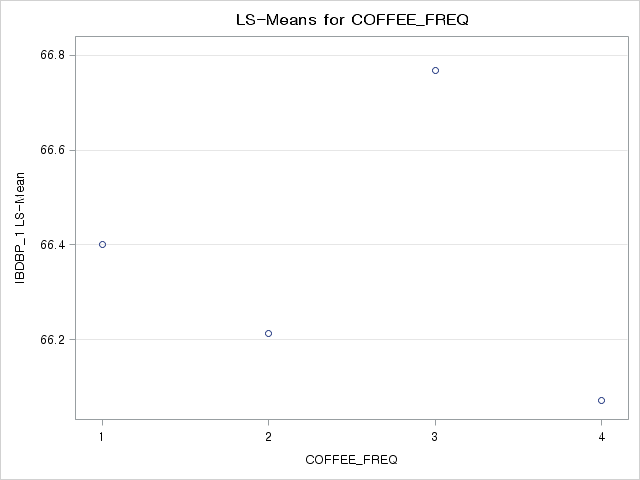

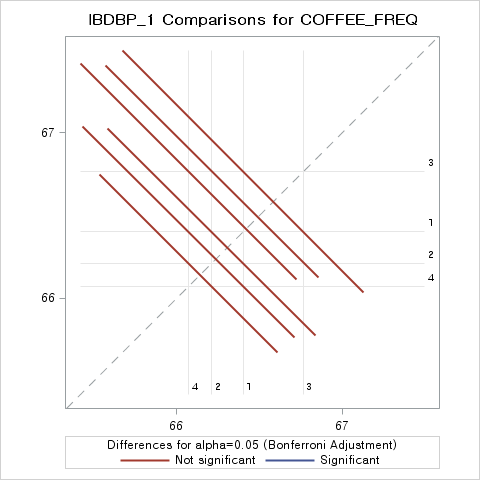


**Variable: Body mass index**


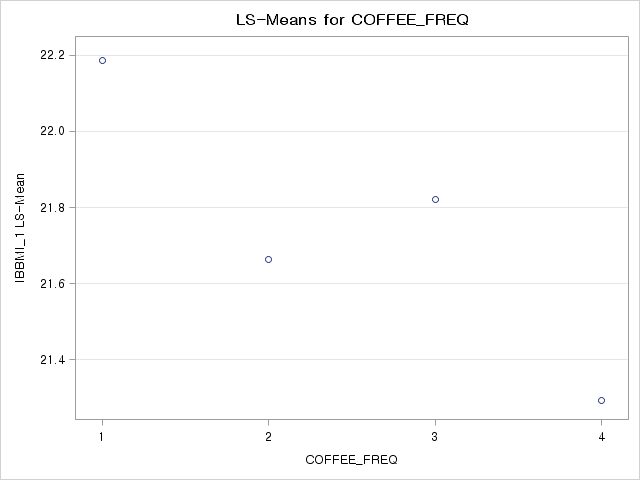

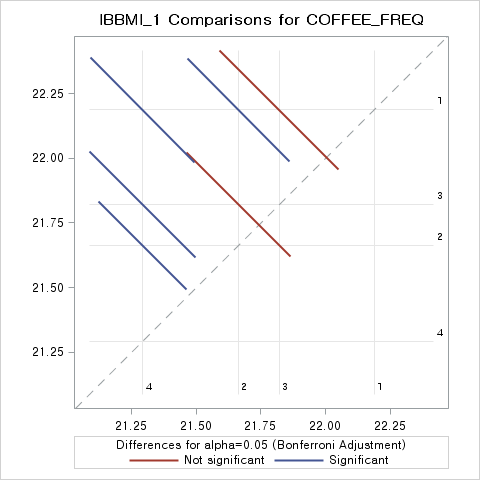


**Variable: Parity**


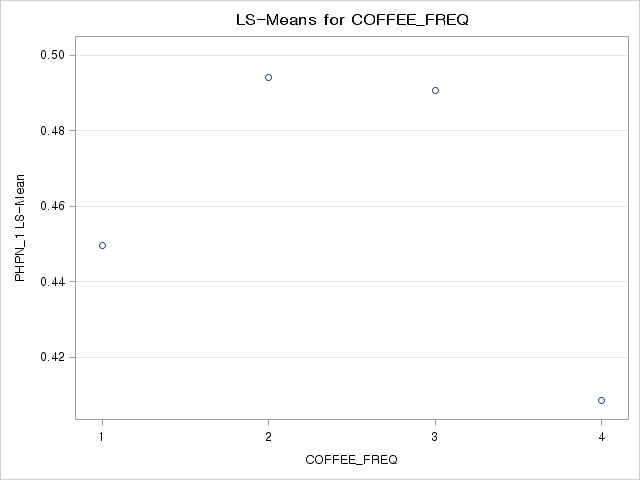

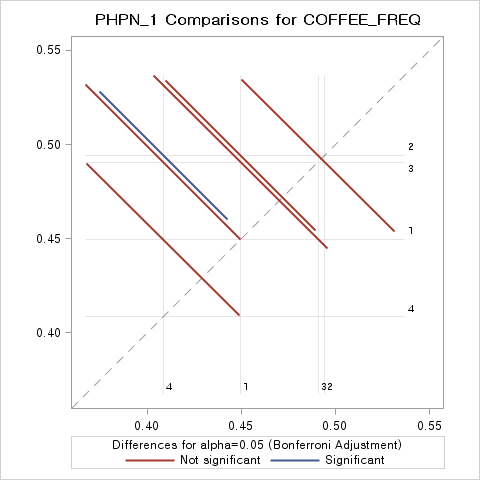

Supplement: Supplementary file 5 — Supplementary Fig. 2. Post-hoc analysis using the Bonferroni test [file 12884_2020_2798_MOESM5_ESM.docx]
