# Supplementary material for: Maternal coffee intake and the risk of bleeding in early pregnancy: a cross-sectional analysis
Source: BMC Pregnancy Childbirth. 2020 Feb 21;20:121. doi: 10.1186/s12884-020-2798-1 (PMC7035749; doi:10.1186/s12884-020-2798-1)
Supplement: Supplementary file 6 — Supplementary File 1. Questionnaires for the KPOS study [file 12884_2020_2798_MOESM6_ESM.docx]

**Questionnaires for the KPOS Study**

*The Korean Pregnancy Outcome study (KPOS)*

Examination date: - - (YYYY-MM-DD)

ID: PIC- -

Date of birth: - - (YYYY-MM-DD)

Date of consent to participation: - - (YYYY-MM-DD)

Gestation weeks: - (WW-DD)

**[Socio-demographics]**

| **Socio-demographics** | **Unit** |
| --- | --- |
| Height | cm |
| Current weight | __ _.__ kg |
| Pre-pregnancy weight | __ _.__ kg/m^2^ |
| Current BMI | __ _.__ kg |
| Pre-pregnancy BMI | __ _.__ kg/m^2^ |
| Systolic blood pressure | mmHg |
| Diastolic blood pressure | mmHg |
| Marital status | ①Never married ②Cohabited ③Married  ④Separated ⑤Divorced ⑥Widowed |
| Job status | ①Yes ②No |
| └ *If you have a job, what type of employment do you have?* | |
|  | ①Self-employed or Employer ②Wage worker  ③Unpaid family worker |
| └ *If you are a wage worker, what is your position* | |
|  | ①Full-time worker ②Part-time worker ③Daily worker |
| Educational status | ①Uneducated ②Elementary school ③Middle school  ④High school ⑤College or University ⑥Graduate school |
| Household income  (1,000 Korean won / month) | ①<1,000 ②1,000-2,000 ③2,000-3,000  ④3,000-4,000 ⑤4,000-5,000 ⑥≥5,000 |

**[Health behavior]**

| **Health behavior** | **Unit** |
| --- | --- |
| Cigarette smoking | ①Never smoker ②Former smoker ③Current smoker |
| └ *If you are a former smoker, when did you quit?* | |
|  | ①Before pregnancy ②Before 6 weeks of gestation  ③6 – 10 weeks of gestation worker ④after 10 weeks of gestation |
| └ *If you are a former or current smoker, how much did you smoking?* | |
|  | piece(s) / day |
| └ *If you are a former or current smoker, how long did you smoking?* | |
|  | year month |
| Passive smoking | ①Yes ②No |
| Alcohol drinking | ①Never drinker ②Former drinker ③Current drinker |
| └ *If you are a former drinker, when did you quit drinking?* | |
|  | ①Before pregnancy ②Before 6 weeks of gestation  ③6 – 10 weeks of gestation worker ④after 10 weeks of gestation |
| └ *If you are a former or current drinker, how much did you drinking?* | |
|  | glass(es) / once |
| └ *If you are a former or current drinker, how long did you drinking?* | |
|  | ①≤1/month ②2-4/month ③2-3/week ④≥4/week ⑤Daily |

**[Physical activity]**

| **Pre-pregnancy** | **Unit** |
| --- | --- |
| Major physical activity | ①Static activity ②Light activity ③Moderate activity  ④Vigorous activity ⑤Strong activity |
| └ *Did you have a vigorous activity before pregnancy?* | |
|  | ①Yes ( day / week, hour(s) minute(s))  ②No |
| └ *Did you have a moderate activity before pregnancy?* | |
|  | ①Yes ( day / week, hour(s) minute(s))  ②No |
| └ *Did you go out for walking before pregnancy?* | |
|  | ①Yes ( day / week, hour(s) minute(s))  ②No |
| └ *Did you have a resistance exercise before pregnancy?* | |
|  | ①Yes ( day / week, hour(s) minute(s))  ②No |
| **Current pregnancy** | **Unit** |
| Major physical activity | ①Static activity ②Light activity ③Moderate activity  ④Vigorous activity ⑤Strong activity |
| └ *Did you have a vigorous activity after pregnancy?* | |
|  | ①Yes ( day / week, hour(s) minute(s))  ②No |
| └ *Did you have a moderate activity after pregnancy?* | |
|  | ①Yes ( day / week, hour(s) minute(s))  ②No |
| └ *Did you go out for walking after pregnancy?* | |
|  | ①Yes ( day / week, hour(s) minute(s))  ②No |
| └ *Did you have a resistance exercise after pregnancy?* | |
|  | ①Yes ( day / week, hour(s) minute(s))  ②No |

**[Dietary pattern]**

| **Dietary pattern before pregnancy** | **Unit** |
| --- | --- |
| Breakfast | ①Everyday ②5-6/week ③5-6/week ④5-6/week ⑤Seldom |
| Lunch | ①Everyday ②5-6/week ③5-6/week ④5-6/week ⑤ Seldom |
| Dinner | ①Everyday ②5-6/week ③5-6/week ④5-6/week ⑤ Seldom |
| Snack | ①Everyday ②5-6/week ③5-6/week ④5-6/week ⑤ Seldom |
| Eating out | ①Everyday ②5-6/week ③5-6/week ④5-6/week ⑤ Seldom |
| Late-night meal | ①Everyday ②5-6/week ③5-6/week ④5-6/week ⑤ Seldom |
| Preference for taste | ①Salty ②Sweet ③Sour ④Spicy ⑤Greasy |
| Coffee intake | ①≥2 cups/day ②1 cup/day ③4-5 cup/week ④2-3 cup/week ⑤Seldom |
| └ *If you are a regular coffee drinker, check the preference type of coffee?* | |
|  | ①Black coffee  ②Black coffee with sugar  ③Black coffee with non-dairy creamer  ④Instant coffee with sugar and non-dairy creamer |
| Multigrain rice | ①3/day ②2/day ③1/day ④4-6/week ⑤2-3/week ⑥1/week ⑦Seldom |
| High-fat meat | ①3/day ②2/day ③1/day ④4-6/week ⑤2-3/week ⑥1/week ⑦Seldom |
| Fish | ①3/day ②2/day ③1/day ④4-6/week ⑤2-3/week ⑥1/week ⑦Seldom |
| Bean or Tofu | ①3/day ②2/day ③1/day ④4-6/week ⑤2-3/week ⑥1/week ⑦Seldom |
| Pickled vegetables or Salted seafood | ①3/day ②2/day ③1/day ④4-6/week ⑤2-3/week ⑥1/week ⑦Seldom |
| Instant food | ①3/day ②2/day ③1/day ④4-6/week ⑤2-3/week ⑥1/week ⑦Seldom |
| Soda | ①3/day ②2/day ③1/day ④4-6/week ⑤2-3/week ⑥1/week ⑦Seldom |
| Snack | ①3/day ②2/day ③1/day ④4-6/week ⑤2-3/week ⑥1/week ⑦Seldom |
| Ramen | ①3/day ②2/day ③1/day ④4-6/week ⑤2-3/week ⑥1/week ⑦Seldom |
| Marine algae | ①3/day ②2/day ③1/day ④4-6/week ⑤2-3/week ⑥1/week ⑦Seldom |
| Fried foods | ①3/day ②2/day ③1/day ④4-6/week ⑤2-3/week ⑥1/week ⑦Seldom |
| Fruits | ①3/day ②2/day ③1/day ④4-6/week ⑤2-3/week ⑥1/week ⑦Seldom |
| Vegetables | ①3/day ②2/day ③1/day ④4-6/week ⑤2-3/week ⑥1/week ⑦Seldom |
| Dairy foods | ①3/day ②2/day ③1/day ④4-6/week ⑤2-3/week ⑥1/week ⑦Seldom |

**[History of diseases]**

| **Name of diseases** | **Existence of diseases** | **Date of diagnosis**  **(YYYY)** | **Usage of medication** | **Name of medication** |
| --- | --- | --- | --- | --- |
| Hypertension | ①Yes ②No ③Not sure | **________** | ①Yes ②No | **________** |
| Diabetes mellitus | ①Yes ②No ③Not sure | **________** | ①Yes ②No | **________** |
| Thyroid insufficiency | ①Yes ②No ③Not sure | **________** | ①Yes ②No | **________** |
| Congenital heart disease | ①Yes ②No ③Not sure | **________** | ①Yes ②No | **________** |
| Asthma | ①Yes ②No ③Not sure | **________** | ①Yes ②No | **________** |
| Chronic kidney disease | ①Yes ②No ③Not sure | **________** | ①Yes ②No | **________** |
| Auto-immune disease | ①Yes ②No ③Not sure | **________** | ①Yes ②No | **________** |
| Atopic dermatitis | ①Yes ②No ③Not sure | **________** | ①Yes ②No | **________** |
| Depression | ①Yes ②No ③Not sure | **________** | ①Yes ②No | **________** |
| Epilepsy | ①Yes ②No ③Not sure | **________** | ①Yes ②No | **________** |
| Polycystic ovarian syndrome | ①Yes ②No ③Not sure | **________** | ①Yes ②No | **________** |
| Hepatitis type A | ①Yes ②No ③Not sure | **________** | ①Yes ②No | **________** |
| Hepatitis type B | ①Yes ②No ③Not sure | **________** | ①Yes ②No | **________** |
| Tuberculosis | ①Yes ②No ③Not sure | **________** | ①Yes ②No | **________** |
| Mental disorders  (except depression) | ①Yes ②No ③Not sure | **________** | ①Yes ②No | **________** |
| Etc (______ __) | ①Yes ②No ③Not sure | **________** | ①Yes ②No | **________** |

**[Family history of diseases]**

| **Name of diseases** | **Existence of diseases** | **Related family** |
| --- | --- | --- |
| Hypertension | ①Yes ②No ③Not sure | ①Grandfather ②Grandmother  ③Father ④Mother ⑤Sibling |
| Diabetes mellitus | ①Yes ②No ③Not sure | ①Grandfather ②Grandmother  ③Father ④Mother ⑤Sibling |
| Gestational diabetes mellitus | ①Yes ②No ③Not sure | ①Grandfather ②Grandmother  ③Father ④Mother ⑤Sibling |
| Eclampsia | ①Yes ②No ③Not sure | ①Grandfather ②Grandmother  ③Father ④Mother ⑤Sibling |
| Depression | ①Yes ②No ③Not sure | ①Grandfather ②Grandmother  ③Father ④Mother ⑤Sibling |
| Mental disorders  (except depression) | ①Yes ②No ③Not sure | ①Grandfather ②Grandmother  ③Father ④Mother ⑤Sibling |

**[Reproductive and Birth history]**

| **Reproductive history** | **Unit** |
| --- | --- |
| Gravidity (No) | **________** time(s) |
| Pregnancy (No) | **________** time(s) |
| Living child (No) | **________** person(s) |
| Death child (No) | **________** person(s) |
| Artificial abortion (No) | **________** time(s) |
| Spontaneous abortion (No) | **________** time(s) |

| **Birth history** | **Unit** |
| --- | --- |
| Number of birth | **________** time(s) |
| Number of fetus | ①Singleton ②Twin or more |
| Date of birth | **________** year |
| Gestation weeks | **________** weeks |
| Birth weight | **_** _._**___** kg |
| Delivery mode | ①Vaginal ②Cesarean |
| History of gestational diabetes mellitus | ①Yes ②No |
| History of hypertensive disorders of pregnancy | ①Yes ②No |
| History of antenatal depression | ①Yes ②No |
| History of postpartum depression | ①Yes ②No |
| History of mental disorders during pregnancy | ①Yes ②No |

**[Current pregnancy information]**

| **Current pregnancy information** | **Unit** |
| --- | --- |
| Last normal menstrual period | - - (YYYY-MM-DD) |
| Expected of delivery date | - - (YYYY-MM-DD) |
| Method of conception | ①Normal ②Ovulation induction  ③Artificial insemination ④In vitro fertilization |
| Number of fetus | ①Singleton ②Twin |
| Emesis | ①Yes ②No |
| └ *If you have an emesis during pregnancy, check the severity of your status.* | |
|  | ①Mild ②Severe ③Inpatient treatment |
| Bleeding in early pregnancy | ①Yes ②No |
| └ *If you have a bleeding in early pregnancy, check the severity of your status.* | |
|  | ①Stabilization ②Drug treatment ③Inpatient treatment |
| Supplement intake |  |
| └ *Multivitamin* | ①Yes ②No ①Pre-pregnancy ②After pregnancy ___weeks |
| └ *Folic acid* | ①Yes ②No ①Pre-pregnancy ②After pregnancy ___weeks |
| └ *Iron* | ①Yes ②No ①Pre-pregnancy ②After pregnancy ___weeks |
| └ *Calcium* | ①Yes ②No ①Pre-pregnancy ②After pregnancy ___weeks |
| └ *Multivitamin* | ①Yes ②No ①Pre-pregnancy ②After pregnancy ___weeks |
| └ *Oriental medicine* | ①Yes ②No ①Pre-pregnancy ②After pregnancy ___weeks |
| └ *etc* | ①Yes ②No ①Pre-pregnancy ②After pregnancy ___weeks |

**[Laboratory values]**

| **Laboratory values** | **Result** | **Unit** |
| --- | --- | --- |
| Hb |  | g/dL |
| Hct |  | % |
| PLT |  | x10^3^/uL |
| WBC |  | x10^3^/uL |
| Glucose |  | mg/dL |
| AST |  | IU/L |
| ALT |  | IU/L |
| BUN |  | mg/dL |
| Creatinine |  | mg/dL |
| Total protein |  | g/dL |
| Albumin |  | g/dL |
| Cholesterol |  | mg/dL |

**[Results of pregnancy]**

Examination date: - - (YYYY-MM-DD)

| **Results of mother** | **Unit** | |
| --- | --- | --- |
| Gestation weeks of delivery | - (WW-DD) | |
| Pregnancy result | ①Miscarriage or Abortion ②Stillbirth  ③Pre-term delivery ④Full-term delivery | |
| Height | Cm | |
| Current weight | __ _.__ kg | |
| Current BMI | __ _.__ kg | |
| Systolic blood pressure | mmHg | |
| Diastolic blood pressure | mmHg | |
| Delivery mode | ①Vaginal ②Cesarean | |
| Complications at delivery | ①Yes ②No | |
| └ *If you have complications at delivery, what type of complications did you have?* | | |
|  | ①Injuries of parturient canal ②Abruption placenta  ③Premature rupture of membranes ④Other | |
| **Results of fetus** | **Unit** | |
| Number of live births | ①Singleton ②Twin | |
|  | **1^st^ baby** | **2^nd^ baby** |
| Sex | ①Boy ②Girl | ①Boy ②Girl |
| Height | . Cm | . Cm |
| Weight | g | g |
| Head Circumference | . cm | . cm |
| APGAR score | 1 minute:  5 minutes: | 1 minute:  5 minutes: |
| Neonatal intensive care unit | ①Yes ②No | ①Yes ②No |
| Endotracheal intubation | ①Yes ②No | ①Yes ②No |
| Convulsions of newborn | ①Yes ②No | ①Yes ②No |
| Bilirubin value | . mg/dL | . mg/dL |
| Congenital malformation | ①Yes ②No | ①Yes ②No |
|  | □Head and Neck  □Eye  □ENT  □Lungs  □Heart  □Abdomen  □Kidney  □Genitalia  □Thorax  □Skeletal (trunk, spine)  □Neuto  □Others ( ) | □Head and Neck  □Eye  □ENT  □Lungs  □Heart  □Abdomen  □Kidney  □Genitalia  □Thorax  □Skeletal (trunk, spine)  □Neuto  □Others ( ) |
